# Mutation in Genes Encoding Key Functional Groups Additively Increase Mortality in Patients with *BRAF^V600E^*-Mutant Advanced Papillary Thyroid Carcinoma

**DOI:** 10.3390/cancers13225846

**Published:** 2021-11-22

**Authors:** Eyun Song, Meihua Jin, Ahreum Jang, Min Ji Jeon, Dong Eun Song, Hye Jin Yoo, Won Bae Kim, Young Kee Shong, Won Gu Kim

**Affiliations:** 1 Division of Endocrinology and Metabolism, Department of Internal Medicine, Korea University College of Medicine and School of Medicine, Seoul 08308, Korea; eyunsong@gmail.com (E.S.); deisy21@korea.ac.kr (H.J.Y.); 2 Division of Endocrinology and Metabolism, Department of Internal Medicine, Asan Medical Center, University of Ulsan College of Medicine, Seoul 05505, Korea; meihua711@naver.com (M.J.); ahreumjang@gmail.com (A.J.); mj080332@gmail.com (M.J.J.); kimwb@amc.seoul.kr (W.B.K.); ykshong@amc.seoul.kr (Y.K.S.); 3 Department of Pathology, Asan Medical Center, University of Ulsan College of Medicine, Seoul 05505, Korea; hipuha@hanmail.net

**Keywords:** papillary thyroid carcinoma, genetic mutation, BRAF, functional groups

## Abstract

**Simple Summary:**

The *BRAF^V600E^* point mutation is the most common driver mutation in papillary thyroid carcinoma (PTC) and is known to be associated with aggressive clinical features. However, the negative prognostic impact of *BRAF^V600E^* on PTC mostly depends on tumor characteristics, not on itself. Moreover, the prognosis of *BRAF^V600E^*-mutant PTCs varies widely implying the genetic diversity of this subtype. Additional genetic alterations other than *BRAF^V600E^* may be responsible for the aggressiveness of this group but to date, no mutations other than *TERT* promoter mutation have been identified. This study aimed to investigate the effect of additional genetic alterations, focusing on the mutations in genes encoding functional groups on survival in *BRAF^V600E^*-mutant PTCs. We observed that coexistence of mutations in *BRAF^V600E^* and the three functional groups had the worst survival in patients with PTCs compared with mutations in *BRAF^V600E^* and genes other than those associated with functional groups or mutations in only *BRAF^V600E^*.

**Abstract:**

The prognosis of *BRAF^V600E^*-mutant papillary thyroid carcinoma (PTC) ranges from indolent to highly aggressive courses. To better define the genetic diversity of this subtype, we evaluated the survival according to the presence of an additional mutation in genes encoding functional groups (FGs) in *BRAF^V600E^*-mutant advanced PTC patients. Targeted next-generation sequencing was performed in primary tumors of 50 *BRAF^V600E^*-mutant PTCs with distant metastasis or aggressive variants. The mutation in genes encoding FGs included alterations in histone methyltransferases, SWI/SNF subunit, and the PI3K/AKT/mTOR pathway. The primary outcome was overall survival (OS). Fifteen patients only had the *BRAF^V600E^*-mutation (group 1), 22 had *BRAF^V600E^* and mutation other than FGs (group 2), and 13 had *BRAF^V600E^* and FG mutation (group 3). OS was significantly lower in patients with FG mutations (*p* = 0.001) than those without, and group 3 patients had the worst survival (*p* = 0.004). OS significantly varied among none, one, or two FG mutation sites (*p* = 0.005). Presence of FG mutation was independently associated with increased mortality (hazard ratio 11.65, 95% confidence interval 1.39–97.58, *p* = 0.024). Coexistence of mutations in *BRAF^V600E^* and genes encoding FGs was associated with high mortality. Identification of FG mutation in *BRAF^V600E^*-mutant PTCs may be valuable in risk stratifying this subtype.

## 1. Introduction

Remarkable progress has been made in the genomic landscape of thyroid cancers over the last decade [1,2,3]. Papillary thyroid carcinoma (PTC), formerly considered to be a single entity, is now regarded as a complex of several tumor types harboring mutually exclusive activating mutations of genes encoding effectors that signal through the mitogen-activated protein kinase (MAPK) pathway [4]. The *BRAF^V600E^* point mutation is the most common one, accounting for 60% of these mutations [1]. This mutation is known to be associated with aggressive clinical features and increased mortality [5,6]. Yet, most PTCs, the majority of which have *BRAF^V600E^* mutations, are indolent and the overall prognosis is fundamentally excellent with only approximately 10% of patients having recurrence or death [2,7,8,9]. Moreover, the negative prognostic impact of *BRAF^V600E^* on PTC mostly depends on tumor characteristics, not on itself [1,6]. These facts imply that considerable clinical variety exists in *BRAF^V600E^*-mutant PTCs and that additional genetic alterations in these tumors may be responsible for the aggressiveness of this group. In fact, coexistence of *BRAF^V600E^* and the telomerase reverse transcriptase (*TERT*)-promotor mutation is known to be strongly associated with aggressive subgroups of PTCs [1], but otherwise, no other genetic events have been validated in risk stratifying *BRAF^V600E^*-mutant PTCs.

Recently, Pappa et al. reported that additional PIK3/AKT/mTOR alterations in PTCs harboring the *BRAF^V600E^* mutation were associated with increased disease-specific mortality. The PIK3/AKT/mTOR pathway is one of the three key functional groups (FGs) presented by Landa et al. in their study of extensive genetic characterization of poorly differentiated thyroid carcinoma (PDTC) and anaplastic thyroid carcinoma (ATC) [10]. Two other key FGs are the histone methyltransferases (HMTs) and the SWItch/Sucrose NonFermenting (SWI/SNF) chromatin remodeling complex, and mutations in these FGs are reported to be more frequent in advanced forms of thyroid cancers than in classic PTCs [11,12], albeit their clinical prognostic significance has not been established.

To better define the additional prognostic role of all three FGs in patients with *BRAF^V600E^*-mutant PTCs, we evaluated survival according to the presence and extent of mutations in FGs in patients with advanced PTCs, defined as PTCs with distant metastases or with aggressive variants, harboring the *BRAF^V600E^* mutation.

## 2. Methods

### 2.1. Patients and Tissue Samples

Tissue samples were collected from primary tumors of 50 patients with *BRAF^V600E^*-mutant advanced PTCs, including PTCs with distant metastasis and aggressive variants of PTC (tall-cell and columnar-cell). Patients with follicular thyroid cancer were excluded because they harbored no *BRAF^V600E^* mutation, and those with PDTC or ATC were also excluded. All samples were examined by one experienced endocrine pathologist (D.E.S). Informed consent was obtained from each patient. The Institutional Review Board of the Asan Medical Center approved all data collection and subsequent analyses.

### 2.2. DNA Extraction and Preparation

Appropriate tissue blocks were selected by the pathologist (D.E.S) for the isolation of DNA from formalin-fixed, paraffin-embedded samples. The subsequent processes of DNA extraction and preparation were the same as used in our previous study [11]. Each specimen’s genomic DNA was isolated from 2–5-μm-thick slices. PicoGreen and NanoDrop (Thermo Fisher Scientific, Waltham, MA, USA) were used to quantify and qualify DNA according to the manufacturer’s methods. To generate standard exome capture libraries, the Agilent SureSelect Target Enrichment protocol (version B.3, Agilent Technologies, Santa Clara, CA, USA) was used. Briefly, 1 µg of DNA was fragmented by adaptive focused acoustic technology (Covaris Inc., Woburn, MA, USA). End repair, A-tailing, and ligation with Agilent adapters were used to create a DNA library. For capturing the exome, we hybridized 250 ng of the DNA library by using SureSelect exome capture baits. Following amplification of the captured DNA, the qPCR Quantification Protocol Guide (Illumina, San Diego, CA, USA) and the TapeStation DNA ScreenTape (Agilent Technologies, Santa Clara, CA, USA) were used to quantify and qualify, respectively, the final purified product.

### 2.3. Targeted Next-Generated Sequencing (NGS) and Analysis Process

Targeted NGS was performed with a total of 50 genes considered to cause relatively frequent mutations in thyroid cancers [13,14] (Table S1). The HiSeq^TM^ 2500 platform (Illumina, San Diego, CA, USA) was used for sequencing. The Burrows–Wheeler Aligner was used to initially map the sequenced reads onto the human reference genome (NCBI build 37) before analysis (version 0.7.12, Sourceforge, San Diego, CA, USA). The Picard tools (version 1.130, Broad Institute, Cambridege, MA, USA) were used to eliminate polymerase chain reaction duplicates. De-duplicated reads were locally realigned with the Genome Analysis Toolkit (GATK version 3.4.0, Broad Institute, Cambridege, MA, USA). GATK’s Haplotype Caller was used for variant genotyping for each sample, which were then annotated by SnpEff (version 4.1g, SnpEff & SnpSift Documentation), dbSNP (version 142, National Center for Biotechnology information, Bethesda, MD, USA), the 1000 genome project (phase3), ClinVar, and ESP6500. Common germline variants or false-positive variants were filtered out manually. Each sample’s mean depth of target lesions ranged from 155.6 to 2705.4.

### 2.4. Functional Groups and Patient Grouping

Three FGs were defined as genes encoding HMTs, the PI3K/AKT/mTOR pathway, and the SWI/SNF chromatin remodeling complex according to previous studies [10,14]. Patients were classified on the basis of the presence and type of additional mutations other than *BRAF*: *BRAF* only (Group 1), *BRAF* with alterations other than FGs (Group 2), and *BRAF* with mutations in FGs (Group 3).

### 2.5. Primary Outcome

The primary outcome of this study was all-cause mortality. Overall survival (OS) (interval from initial surgery to the date of death) was compared among the three different genomic groups.

### 2.6. Statistical Analysis

R version 3.4.0 and the R libraries “survival”, “car”, and “Cairo” (R Foundation for Statistical Computing, Vienna, Austria; http://www.R-project.org, accessed 1 August 2021) were used for data analysis. Continuous variables were presented as medians with interquartile ranges (IQRs) and were analyzed by using the Kruskal–Wallis test. Categorical variables were presented as numbers with percentages and compared by using Pearson’s χ^2^ test. Survival curves were plotted by using the Kaplan–Meier method, and the survival rates among groups were compared by the log-rank test. Cox proportional hazard models were used to evaluate the impact of mutations in FGs on the survival adjusting for age, sex, tumor size, multifocality, extrathyroidal extension, lymph node metastasis, and distant metastasis.

## 3. Results

### 3.1. Clinical Characteristics of Patients

Table 1 summarizes the baseline characteristics of the total 50 patients with advanced PTCs harboring the *BRAF^V600E^* mutation. There were 19 (38.0%) patients with classic PTC, 23 (46.0%) with tall-cell variant PTC, and 8 (16.0%) with columnar-cell variant PTC. Among these patients, 37 (74.0%) had no mutations in function groups―only *BRAF^V600E^*-mutant (group 1) or with oncogenic mutations other than FGs (group 2)—and 13 (26.0%) had mutations in one or more FGs (group 3). The most common pathology type in the patients with no FG mutations were tall-cell variant PTC, whereas classic PTC accounted for 69.2% of the patients with FG mutations. The patients with FGs mutations were significantly older than their counterparts, but there were no differences in sex, primary tumor size, presence of extrathyroidal extension, multifocality, or lymph node metastases between the two groups. However, the rate of distant metastases was significantly higher in patients with FG mutations (*p* = 0.017); synchronous distant metastases (15.4% vs. 10.8%) and metachronous distant metastases (53.8% vs. 16.2%) were both more frequent in the FG. During the mean 7.5 years of follow-up, 11 (22.0%) patients died. Among the 11 patients, 9 died due to disease progression of thyroid cancer while the exact reason for death of the other two is not clear as they were lost of follow-up from our hospital. Higher rate of mortality was observed in FG mutation group with the rate of 8.1% in patients without FG mutation and 61.5% in patients with FG mutation (*p* < 0.001).

### 3.2. Mutational Profile

**Figure 1:**
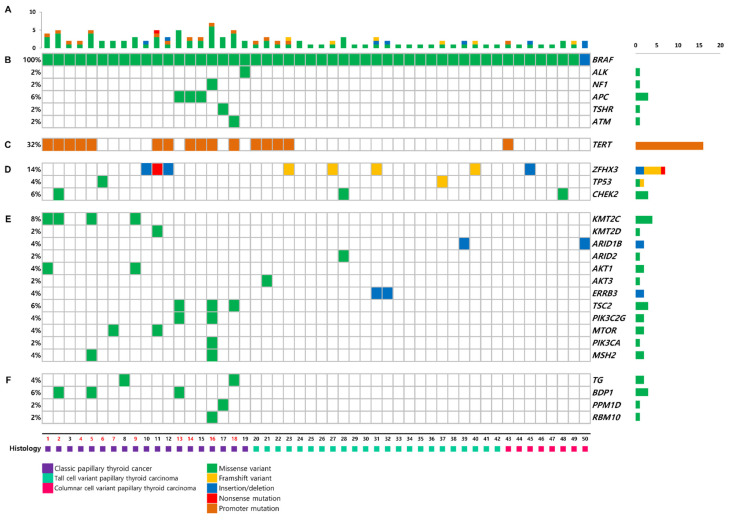
Mutations in 50 primary tumors of BRAF^V600E^-mutant PTCs. (**A**) Numbers of mutations, (**B**) driver genes, (**C**) TERT promoter, (**D**) tumor suppressor genes, (**E**) key pathways and functional groups, and (**F**) other genes. Sample numbers in red letters indicate patients who died during follow-up.

Median of 3 (IQR 2–4) somatic mutations were detected by targeted NGS (Figure 1A). In addition to *BRAF^V600E^* mutation, other driver mutations observed were APC (6%), ALK (2%), NF1 (2%), TSHR (2%), and ATM (2%) (Figure 1B). TERT promoter mutation was identified in 32% (16/50) of the patients (Figure 1C). C228T (10/16, 63%) was the most common mutation, followed by C250T (6/16, 37%). ZFHX3, the tumor suppressor gene, was mutated in 14% of the patients (Figure 1D). Mutations in HMTs, KMT2C, and KMT2D were present in 10% (Figure 1E). Genes encoding SWI/SNF chromatin remodeling complex were altered in 6%: 2% in ARID1B, and 4% in ARID2. Mutations in genes encoding the members of PI3K/AKT/mTOR pathway were detected in 16% of the patients, including PIK3CA, PIK3C2G, AKT1, AKT3, TSC2, and MTOR. Other alterations are shown in Figure 1F.

### 3.3. Survival in Different Genomic Subgroups

**Figure 2:**
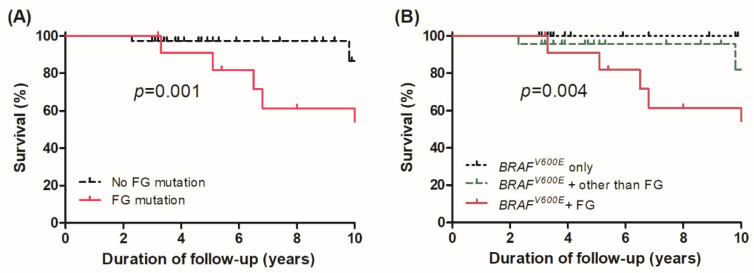
Overall survival curve (**A**) of patients with advanced PTC according to presence of mutations in functional groups and (**B**) among three genomic subgroups: *BRAF^V600E^* only (group 1) vs. *BRAF^V600E^* + additional mutations other than functional groups (group 2) vs. *BRAF^V600E^* + mutations in functional groups (group 3).

OS was compared according to the presence of FG mutations. As shown in Figure 2A, OS was significantly lower in patients with FG mutations than in those without (hazard ratio [HR] 6.96, 95% confidence interval [CI] 1.88–25.74, *p* = 0.001). When compared among the three pre-specified genomic subgroups, there were also significant differences in OS in groups 1, 2, and 3 (Figure 2B, log-rank *p* = 0.004); patients with *BRAF^V600E^* and additional mutations in FGs (group 3) had the poorest survival.

**Figure 3:**
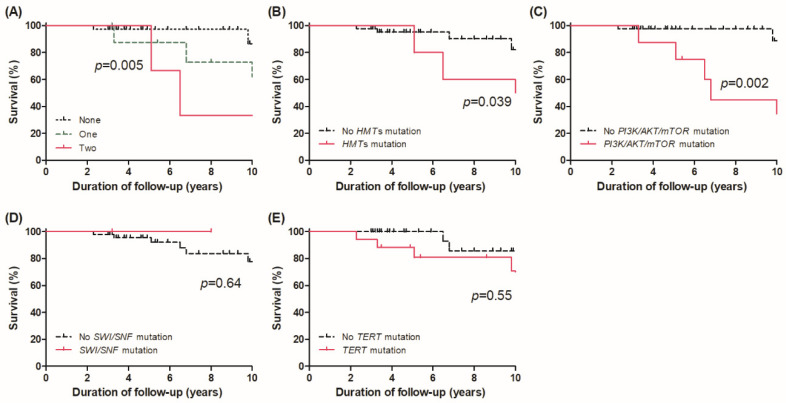
Overall survival curves according to (**A**) the number of mutation sites in functional groups, presence of mutations in (**B**) HMTs, (**C**) PI3K/AKT/mTOR pathway, (**D**) SWI/SNF chromatin remodeling complex, and (**E**) *TERT* promoter.

Figure 3A shows the survival curves according to the numbers of FG mutation sites; mutations in none vs. one vs. two FGs. Significant differences in OS were observed among the three groups, and patients with oncogenic mutations in two of the FGs had the lowest OS (log-rank *p* = 0.005). When assessing the OS on the basis of the presence of each alteration of FGs, OS was poorer for patients with mutations in HMTs and the PI3K/AKT/mTOR pathway than for those without (*p* = 0.039 and *p* = 0.002, Figure 3B and Figure 3C, respectively). However, presence of mutation in SWI/SNF subunits did not show differences in OS (*p* = 0.064, Figure 3D). Moreover, the presence of *TERT* promoter mutation was not associated with OS (*p* = 0.55, Figure 3E).

**Figure 4:**
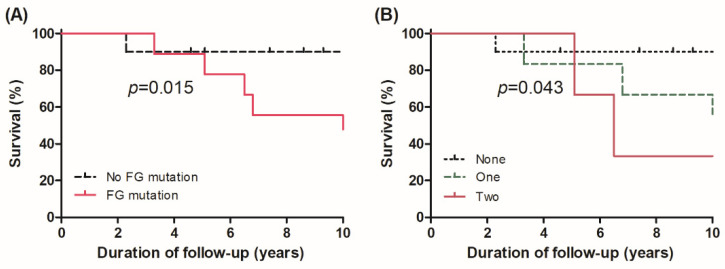
Overall survival curve in patients with classic PTC according to (**A**) presence of mutation in functional groups and (**B**) the number of mutation sites in functional groups.

Subgroup analysis was performed with patients with classic PTC only (*n* = 19) and the results were largely similar. There was a significant difference in OS according to the presence of FG mutation (*p* = 0.015, Figure 4A) and the numbers of FG mutation sites (*p* = 0.043, Figure 4B). 

### 3.4. Effect of Alterations in FGs on Survival

Multivariate analyses were performed to evaluate the effect of mutations in FGs on survival (Table 2). Presence of FG mutation independently increased the risk of mortality, with an adjusted HR of 11.65 (95% CI 1.39–97.58, *p* = 0.024). With no FG mutations as a reference, the presence of mutation at one of the three FGs was associated with a higher risk of mortality, with an adjusted HR of 11.53 (95% CI 2.69–49.4, *p* = 0.001), and mutations at two of the three FGs were associated with the highest risk, with an adjusted HR of 60.12 (95% CI 10.63–340.2, *p* < 0.001). A comparison of the three groups showed that group 3 had a higher risk of mortality than group 1 (HR 13.96, 95% CI 1.67–116.9, *p* = 0.015), whereas group 2 was not associated with lower survival than group 1 (HR 1.20, 95% CI 0.14–10.04, *p* = 0.867).

## 4. Discussion

This study used targeted NGS to investigate OS according to the genetic landscape of advanced PTCs harboring the *BRAF^V600E^* mutation. We identified that one or more mutations in genes encoding HMTs, the PI3K/AKT/mTOR pathway, and the SWI/SNF chromatin remodeling complex in addition to the *BRAF^V660E^* mutation were associated with increased mortality in patients with advanced PTCs. This association was independent of various clinicopathological risk factors. Moreover, patients with two additional mutations in the FGs had poorer OS than those with one, demonstrating the incremental effect of mutations in the FGs on survival.

The *BRAF^V600E^* mutation is the most frequent driver mutation observed in PTCs and has drawn particular attention due to its role in the tumorigenesis of PTCs by activating the MAPK pathway to a greater degree than other drivers, such as *RAS* mutations or *RET* rearrangements [15]. Previous studies have reported the association between the *BRAF^V600E^* mutation and tumor aggressiveness in PTCs, including larger tumor size, higher rates of extrathyroidal extension or lymph node metastases, and increased cancer-related mortality [5,6,16]. Nevertheless, the clinical prognosis of the PTCs with the *BRAF^V600E^* mutation ranges from very indolent to highly aggressive courses, which implies the genetic diversity within this subtype. While remaining reliant on their driver *BRAF^V600E^*, this subtype may evolve to a more aggressive form by the acquisition of additional genetic alteration. To date, *TERT* promoter mutation is the only well-established additional alteration with consistent results among *BRAF^V660E^* mutant PTCs [1]. Our study aimed to identify other additional mutations that is responsible for the aggressiveness in this subtype and thus included only those with *BRAF^V660E^* mutation. We particularly focused on the additive role of mutations in the three FGs and observed that coexistence of mutations in *BRAF^V600E^* and FGs was associated with the worst survival, whereas additional genetic events other than function groups had less effect on the prognosis.

Three FGs, HMTs, the PI3K/AKT/mTOR pathway, and the SWI/SNF complex, were first defined in thyroid cancers by Landa et al. in their study comparing the genomic profiles between PDTC and ATC [10]. In that landmark study, 20% of PDTCs and 61% of ATCs harbored alterations in the above FGs. The same study group explored the FG mutations in their subsequent work, which included 22 well-differentiated thyroid carcinomas as well as 35 PDTC, and provided the feasibility to apply the FG mutation in different subtypes of thyroid cancers including PTCs. We previously reported the frequency of mutations in these FGs, which were 43.7% in PTCs with distant metastasis [11] and 34% in PTCs with aggressive variants [12], whereas in the present study of only *BRAF^V600E^*-driven PTCs, the rate of mutations was 26% [13/50]. The mutational rate in classic PTC from the Cancer Genome Atlas is 20% [1], whereas the rates in advanced PTCs range between those in the classic PTC and ATC, a finding in line with the order of clinical prognosis. However, the investigations of genotype–phenotype correlations regarding the alterations in FGs in PTCs are very limited. A previous study using mouse thyroid cancer cell lines showed that loss of SWI/SNF subunits was related to radioiodine refractoriness and resistant to MAPK inhibitor-based redifferentiation therapies in *BRAF^V600E^*-mutant PTCs, which needs validation in humans [17]. Mutations in PI3K/AKT/mTOR pathway were reported frequently in advanced thyroid cancers (PDTC or ATC) especially in metastatic or recurrent cases, and also in follicular thyroid carcinomas [18,19]. They may promote tumorigenesis and invasiveness and may lead to distant metastasis or radioactive iodine refractoriness. A recent study of 225 patients with *BRAF^V600E^*-driven PTC reported that PI3K/AKT/mTOR alteration in these patients increased disease-specific mortality, and its effect was independent of disease stage [20]. This approach was valuable for improving risk stratification in PTCs with the *BRAF^V600E^* mutation, but was only limited to the PI3K/AKT/mTOR pathway. Our study further assessed the effect of mutations in other FGs as well, and we were able to stratify the study patients into different risk groups by the presence and extent of mutations in these FGs. Further studies are necessary to examine the basis for how *BRAF^V600E^*-mutant PTCs evolve to more aggressive forms by accumulating FG mutations. 

Of note, in this study, the presence of additional *TERT* promoter mutations was not associated with increased mortality in patients with *BRAF^V600E^*-mutant PTC. *TERT* promoter mutations are one of the most potent prognostic biomarkers in thyroid cancer, and it is well known that coexistence of the *TERT* promoter and *BRAF^V600E^* mutation exerts synergistic effects leading to poor prognosis [1]. *TERT* is reactivated by somatic *TERT* alterations or epigenetic modulations that result in development and progression of cancers by lengthening of telomeres [21]. Although our study fell short of showing statistical significance of the oncogenic synergism of *TERT* promoter and *BRAF^V600E^* mutations, possibly because of the small sample size, additional alterations in both the *TERT* promoter and genes encoding FGs may serve as important genetic patterns with prognostic implications for patients with *BRAF^V600E^*-mutant PTCs.

A major limitation of this study was the small number of patients. Aggressive variants were also limited to the tall-cell and columnar-cell variants, and other subtypes were not available. Subgroup analysis by excluding patients with classic PTC was not feasible due to small number of patients who died during the study period. However, we were able to perform subgroup analysis within the classic PTC subtype, which was the major subtype in patients with FG mutation, and showed significant difference according to the presence of FG mutation. Another major limitation is the low number of events (11 deaths) which leads to low statistical power. Further study with larger sample size and sufficient events are necessary to validate our findings. Lastly, targeted NGS was performed with only 50 genes, so the total accumulation rate of functional genes may have been underestimated. Despite these limitations, the strength of our study was the identification for the first time of the independent prognostic role of additional mutations in all three FGs after adjusting for various clinicopathological factors.

## 5. Conclusions

Coexistence of mutations in *BRAF^V600E^* and FGs (HMTs, the PI3K/AKT/mTOR pathway, and the SWI/SNF chromatin remodeling complex) exhibited the worst survival in patients with PTCs compared with coexistence of mutations in *BRAF^V600E^* and genes other than those associated with FGs or mutations in only *BRAF^V600E^*. Risk stratification of *BRAF^V600E^*-mutant PTCs by identifying additional mutations in FGs can readily select those with aggressive behavior for more individualized treatment. The strong association between FG mutations and survival observed in our study may further provide the basis for development of therapeutic biomarkers in patients with *BRAF^V600E^*-mutant PTCs.

## Figures and Tables

**Table 1:** Baseline characteristics of patients with advanced PTC harboring the BRAFV600E mutation.

Characteristics	Total (*n* = 50)	Mutations in FGs (−) (*n* = 37)	Mutations in FGs (+) (*n* = 13)	*p*-Value
Pathology				0.026
Classic PTC	19 (38.0%)	10 (27.0%)	9 (69.2%)	
TV PTC	23 (46.0%)	20 (54.1%)	3 (23.1%)	
CCV PTC	8 (16.0%)	7 (18.9%)	1 (7.7%)	
Age (years)	49.5 (38.0–59.5)	48.0 (35.0–55.0)	58.9 (48.9–68.4)	0.025
Sex (female)	29 (58.0%)	23 (62.2%)	6 (46.2%)	0.497
Primary tumor size (cm)	2.3 (1.5–3.2)	2.1 (1.4–2.8)	3.0 (1.5–3.8)	0.341
Extrathyroidal extension				0.535
Microscopic	23 (46%)	17 (45.9%)	6 (46.2%)	
Gross	19 (38%)	15 (40.5%)	4 (30.8%)	
Multifocality (yes)	21 (42.0%)	16 (43.2%)	5 (38.5%)	0.801
LN metastases (yes)				0.482
N1a	15 (30.0%)	12 (32.4%)	3 (23.1%)	
N1b	30 (60.0%)	22 (59.4%)	8 (61.5%)	
Distant metastases				0.017
Synchronous	6 (12.0%)	4 (10.8%)	2 (15.4%)	
Metachronous	13 (26.0%)	6 (16.2%)	7 (53.8%)	
AJCC TNM 8th stage				0.248
I	33 (66.0%)	27 (73.0%)	6 (46.2%)	
II	12 (24.0%)	7 (18.9%)	5 (38.5%)	
III	1 (2.0%)	1 (2.7%)	0 (0.0%)	
IV	4 (8.0%)	2 (5.4%)	2 (15.4%)	
Follow-up duration (years)	5.3 (3.4–9.8)	4.9 (3.4–9.3)	6.8 (5.1–13.6)	0.219
Overall mortality	11 (22.0%)	3 (8.1%)	8 (61.5%)	<0.001

Abbreviations: FG, functional-group; PTC, papillary thyroid carcinoma; TV, tall-cell variant; CCV, columnar-cell variant; AJCC TNM, American Joint Committee on Cancer Tumor-Node-Metastasis.

**Table 2:** Prognostic effects of mutations in functional groups on survival in patients with advanced PTCs harboring the *BRAF^V600E^* mutation.

Risk Factors	Adjusted HR (95% CI) *	*p*-Value
Mutation in functional groups		
Presence vs. absence	11.65 (1.39–97.58)	0.024
Number of mutations in functional groups		
One site vs. none	11.53 (2.69–49.4)	<0.001
Two sites vs. none	60.12 (10.63–340.2)	<0.001
Comparisons in groups		
Group 2 vs. Group 1	1.20 (0.14–10.04)	0.867
Group 3 vs. Group 1	13.96 (1.67–116.9)	0.015
Group 3 vs. Group 2	11.65 (1.39–97.58)	0.024

* Adjusted by age, sex, tumor size, multifocality, extrathyroidal extension, lymph node metastasis, distant metastasis. Group 1: *BRAF^V600E^* only; Group 2: *BRAF^V600E^* + additional mutations other than functioning groups; Group 3: *BRAF^V600E^* + mutations in functional groups.

## Data Availability

The data that support the findings of this study are available upon request from the corresponding author.

## Supplementary Materials

The following are available online at https://www.mdpi.com/article/10.3390/cancers13225846/s1, Table S1: List of 50 target genes.
