# Supplementary material for: Mutation in Genes Encoding Key Functional Groups Additively Increase Mortality in Patients with BRAFV600E-Mutant Advanced Papillary Thyroid Carcinoma
Source: Cancers (Basel). 2021 Nov 22;13(22):5846. doi: 10.3390/cancers13225846 (PMC8616313; doi:10.3390/cancers13225846)
Supplement: Supplementary file 1 [file cancers-13-05846-s001.zip › cancers-1477986-supplementary.pdf]

cancers

MDPI

Supplementary Materials: Mutation in Genes Encoding Key Functional Groups Additively Increase Mortality in Patients with *BRAF*^{V600E}-Mutant Advanced Papillary Thyroid Carcinoma

Eyun Song, Meihua Jin, Ahreum Jang, Min Ji Jeon, Dong Eun Song, Hye Jin Yoo, Won Bae Kim, Young Kee Shong and Won Gu Kim

Table S1. List of 50 target genes.

***TERT* promoter**	***BRAF***	***NRAS***	***HRAS***	***KRAS***
EIF1AX	*TP53*	*RB1*	*NF1*	*NF2*
PTEN	*PIK3CA*	*ATM*	*MEN1*	*ARID1B*
TSHR	*MED12*	*RBM10*	*ARID2*	*POLE*
PPM1D	*STK11*	*CHEK2*	*ZFHX3*	*BDP1*
KMT2A	*KMT2C*	*TG*	*EZH1*	*APC*
AKT1	*SPOP*	*ALK*	*RET*	*PIK3C2G*
PIK3CG	*AKT3*	*TSC2*	*MTOR*	*ARID1A*
ARID1B	*ARID5B*	*SMARCB1*	*PBRM1*	*ATRX*
KMT2D	*MSH2*	*FGFR3*	*PDGFRB*	*ERBB3*
